# Bis[2-(diphenyl­phosphanyl-κ*P*)benzaldehyde]­iodidogold(I)

**DOI:** 10.1107/S1600536812011609

**Published:** 2012-03-24

**Authors:** Michael L. Williams, Samuel P. C. Dunstan, Peter C. Healy, Edward R. T. Tiekink

**Affiliations:** a School of Biomolecular and Physical Sciences, Griffith University, Brisbane, Queensland 4111, Australia; b Department of Chemistry, University of Malaya, 50603 Kuala Lumpur, Malaysia

## Abstract

In the title compound, [AuI(C_19_H_15_OP)_2_], the complete mol­ecule is generated by the application of twofold symmetry. The Au^I^ atom is in a trigonal–planar geometry within an IP_2_ donor set with the greatest distortion seen in the P—Au—P angle [128.49 (3) °]. Close intra­molecular Au⋯O inter­actions [3.172 (3) Å] are observed. No specific inter­molecular inter­actions are noted in the crystal packing.

## Related literature

For a discussion on intra­molecular Au⋯O inter­actions, see: Kuan *et al.* (2008 ▶). For related structures, see: Bowmaker *et al.* (1987 ▶); Elsegood *et al.* (2006 ▶).
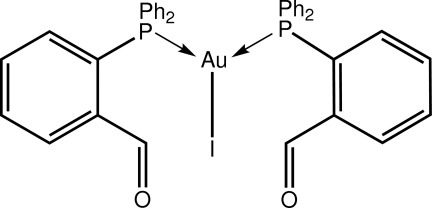


## Experimental

### 

#### Crystal data

[AuI(C_19_H_15_OP)_2_]
*M* *_r_* = 904.43
Monoclinic, 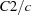

*a* = 18.1099 (13) Å
*b* = 10.1856 (6) Å
*c* = 19.8438 (13) Å
β = 115.965 (2)°
*V* = 3290.9 (4) Å^3^
*Z* = 4
Mo *K*α radiation
μ = 5.54 mm^−1^
*T* = 223 K
0.40 × 0.30 × 0.05 mm

#### Data collection

Bruker SMART CCD diffractometer
Absorption correction: multi-scan (*SADABS*; Bruker, 2000 ▶) *T*_min_ = 0.392, *T*_max_ = 1.000
13430 measured reflections
4792 independent reflections
4319 reflections with *I* > 2σ(*I*)
*R*_int_ = 0.042

#### Refinement

*R*[*F* ^2^ > 2σ(*F* ^2^)] = 0.026
*wR*(*F* ^2^) = 0.059
*S* = 1.01
4792 reflections
200 parameters
H-atom parameters constrained
Δρ_max_ = 1.47 e Å^−3^
Δρ_min_ = −1.34 e Å^−3^

### 

Data collection: *SMART* (Bruker, 2000 ▶); cell refinement: *SAINT* (Bruker, 2000 ▶); data reduction: *SAINT*; program(s) used to solve structure: *SHELXS97* (Sheldrick, 2008 ▶); program(s) used to refine structure: *SHELXL97* (Sheldrick, 2008 ▶); molecular graphics: *ORTEP-3* (Farrugia, 1997 ▶) and *DIAMOND* (Brandenburg, 2006 ▶); software used to prepare material for publication: *publCIF* (Westrip, 2010 ▶).

## Supplementary Material

Crystal structure: contains datablock(s) global, I. DOI: 10.1107/S1600536812011609/su2394sup1.cif

Structure factors: contains datablock(s) I. DOI: 10.1107/S1600536812011609/su2394Isup2.hkl

Additional supplementary materials:  crystallographic information; 3D view; checkCIF report

## Figures and Tables

**Table:** 

Au—I1	2.7188 (3)
Au—P1	2.3200 (6)

**Table:** 

P1—Au—I1	115.755 (16)
P1^i^—Au—P1	128.49 (3)

Symmetry code: (i) 
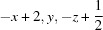
.

**Fig. 1.**
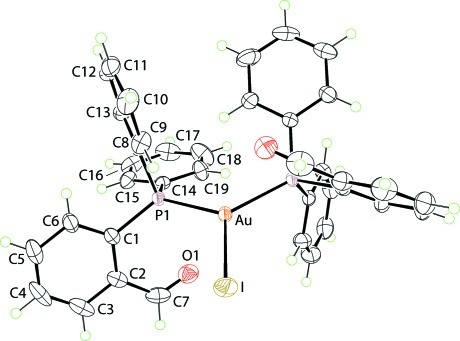
Molecular structure of (I) showing atom-labelling scheme and displacement ellipsoids at the 50% probability level. The molecule has twofold symmetry and unlabelled atoms are related by the symmetry operation 2 - x, y, 1/2 - z.

**Fig. 2.**
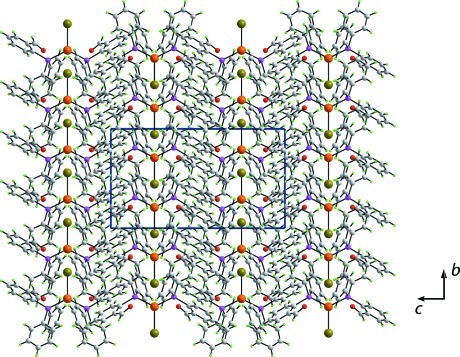
A view in projection down the a axis of the unit-cell contents of (I).

## supplementary crystallographic information

### Comment

The crystal structure of the monophosphinegold(I) chloride complex, (2-CHOC_6_H_4_)Ph_2_PAuCl, where one of the organic substituents on the phosphine has been functionalized with an aldehyde group, has been reported previously (Elsegood *et al.*, 2006). Herein, the crystal structure of the title bis(phosphine)gold(I) iodide analogue (I) is described.

In (I), Fig. 1, the complete molecule is generated by the application of twofold symmetry. The Au atom is in a trigonal planar geometry within a IP_2_ donor set, Table 1, with the greatest distortion manifested in the angle, *i.e*. 128.49 (3)°, subtended by the phosphine ligands, Table 1. The Au—I and Au—P bond lengths in the comparable (Ph_3_P)_2_AuI complex, which also has crystallographic twofold symmetry are 2.754 (1) and 2.333 (2) Å, respectively (Bowmaker *et al.*, 1987); the P—Au—P angle is 132.13 (7)°.

In (I), close intramolecular Au···O interactions of 3.172 (3) Å are noted. Similar interactions of 3.109 (4) and 3.106 (4) Å (two independent molecules) were observed in (2-CHOC_6_H_4_)Ph_2_PAuCl (Elsegood *et al.*, 2006) and their significance has been discussed in the literature (Kuan *et al.*, 2008).

No specific intermolecular interactions are noted in the crystal packing. Globally, molecules are arranged in layers that stack along the *c* axis.

### Experimental

[NBu_4_][AuI_2_] (100 mg, 0.184 mmol) and (2-CHOC_6_H_4_)Ph_2_P (107 mg, 0.368 mmol) were dissolved in warm DMF (10 ml) to give a clear solution. Cooling to room temperature and slow evaporation of solvent yielded clear, colourless crystals of the title complex. *M*.pt: 471–283 K. Analysis: Found C 50.33, H 3.34%. Calculated for C_38_H_30_AuIO_2_P_2_: C 50.46, H 3.34%.

### Refinement

The H atoms were geometrically placed (C—H = 0.94–0.99 Å) and refined as riding with *U*_iso_(H) = 1.2*U*_eq_(C). The maximum and minimum residual electron density peaks of 1.47 and 1.34 e Å^-3^, respectively, were located 0.82 Å and 0.86 Å from the Au atom.

### Crystal data

**Table:**

[AuI(C_19_H_15_OP)_2_]	*F*(000) = 1744
*M**_r_* = 904.43	*D*_x_ = 1.825 Mg m^−^^3^
Monoclinic, *C*2/*c*	Mo *K*α radiation, λ = 0.71069 Å
Hall symbol: -C 2yc	Cell parameters from 7249 reflections
*a* = 18.1099 (13) Å	θ = 2.1–40.5°
*b* = 10.1856 (6) Å	µ = 5.54 mm^−^^1^
*c* = 19.8438 (13) Å	*T* = 223 K
β = 115.965 (2)°	Prism, colourless
*V* = 3290.9 (4) Å^3^	0.40 × 0.30 × 0.05 mm
*Z* = 4	

### Data collection

**Table:**

Bruker SMART CCD diffractometer	4792 independent reflections
Radiation source: fine-focus sealed tube	4319 reflections with *I* > 2σ(*I*)
Graphite monochromator	*R*_int_ = 0.042
ω scans	θ_max_ = 30.0°, θ_min_ = 2.3°
Absorption correction: multi-scan (*SADABS*; Bruker, 2000)	*h* = −25→25
*T*_min_ = 0.392, *T*_max_ = 1.000	*k* = −9→14
13430 measured reflections	*l* = −27→27

### Refinement

**Table:**

Refinement on *F*^2^	Primary atom site location: structure-invariant direct methods
Least-squares matrix: full	Secondary atom site location: difference Fourier map
*R*[*F*^2^ > 2σ(*F*^2^)] = 0.026	Hydrogen site location: inferred from neighbouring sites
*wR*(*F*^2^) = 0.059	H-atom parameters constrained
*S* = 1.01	*w* = 1/[σ^2^(*F*_o_^2^) + (0.0253*P*)^2^] where *P* = (*F*_o_^2^ + 2*F*_c_^2^)/3
4792 reflections	(Δ/σ)_max_ = 0.001
200 parameters	Δρ_max_ = 1.47 e Å^−^^3^
0 restraints	Δρ_min_ = −1.34 e Å^−^^3^

### Special details

**Table:**

Geometry. All s.u.'s (except the s.u. in the dihedral angle between two l.s. planes) are estimated using the full covariance matrix. The cell s.u.'s are taken into account individually in the estimation of s.u.'s in distances, angles and torsion angles; correlations between s.u.'s in cell parameters are only used when they are defined by crystal symmetry. An approximate (isotropic) treatment of cell s.u.'s is used for estimating s.u.'s involving l.s. planes.
Refinement. Refinement of *F*^2^ against ALL reflections. The weighted *R*-factor *wR* and goodness of fit *S* are based on *F*^2^, conventional *R*-factors *R* are based on *F*, with *F* set to zero for negative *F*^2^. The threshold expression of *F*^2^ > 2σ(*F*^2^) is used only for calculating *R*-factors(gt) *etc*. and is not relevant to the choice of reflections for refinement. *R*-factors based on *F*^2^ are statistically about twice as large as those based on *F*, and *R*- factors based on ALL data will be even larger.

### Fractional atomic coordinates and isotropic or equivalent isotropic displacement parameters (Å^2^)

**Table:**

	*x*	*y*	*z*	*U*_iso_*/*U*_eq_	
Au	1.0000	0.290105 (13)	0.2500	0.02140 (5)	
I1	1.0000	0.55703 (3)	0.2500	0.04282 (8)	
P1	1.01880 (4)	0.19113 (6)	0.36168 (3)	0.01989 (13)	
O1	1.17648 (14)	0.2889 (2)	0.38822 (14)	0.0404 (5)	
C1	1.05497 (18)	0.2836 (2)	0.44986 (15)	0.0245 (5)	
C2	1.12816 (18)	0.3569 (3)	0.47629 (17)	0.0329 (6)	
C3	1.1539 (2)	0.4272 (3)	0.5433 (2)	0.0478 (9)	
H3	1.2025	0.4768	0.5609	0.057*	
C4	1.1084 (3)	0.4246 (3)	0.58394 (19)	0.0512 (10)	
H4	1.1263	0.4716	0.6291	0.061*	
C5	1.0371 (2)	0.3533 (3)	0.55821 (16)	0.0430 (8)	
H5	1.0064	0.3510	0.5860	0.052*	
C6	1.0100 (2)	0.2843 (3)	0.49109 (16)	0.0327 (7)	
H6	0.9604	0.2373	0.4735	0.039*	
C7	1.1813 (2)	0.3609 (4)	0.4379 (2)	0.0453 (8)	
H7	1.2227	0.4252	0.4535	0.054*	
C8	0.91749 (15)	0.1302 (3)	0.34615 (13)	0.0218 (5)	
C9	0.8497 (2)	0.2101 (3)	0.30578 (18)	0.0345 (7)	
H9	0.8577	0.2944	0.2908	0.041*	
C10	0.7712 (2)	0.1663 (4)	0.28772 (19)	0.0447 (8)	
H10	0.7262	0.2214	0.2605	0.054*	
C11	0.7575 (2)	0.0440 (4)	0.30874 (18)	0.0414 (8)	
H11	0.7038	0.0160	0.2970	0.050*	
C12	0.8231 (2)	−0.0368 (3)	0.34700 (17)	0.0368 (7)	
H12	0.8142	−0.1213	0.3610	0.044*	
C13	0.90263 (17)	0.0048 (3)	0.36533 (15)	0.0287 (6)	
H13	0.9469	−0.0524	0.3910	0.034*	
C14	1.08135 (16)	0.0436 (2)	0.38691 (14)	0.0212 (5)	
C15	1.10862 (17)	−0.0130 (3)	0.45739 (16)	0.0307 (6)	
H15	1.1002	0.0308	0.4951	0.037*	
C16	1.14815 (18)	−0.1334 (3)	0.47260 (19)	0.0391 (7)	
H16	1.1675	−0.1700	0.5208	0.047*	
C17	1.1591 (2)	−0.1991 (3)	0.4175 (2)	0.0447 (9)	
H17	1.1839	−0.2824	0.4274	0.054*	
C18	1.1339 (2)	−0.1434 (4)	0.3476 (2)	0.0519 (9)	
H18	1.1424	−0.1880	0.3101	0.062*	
C19	1.09597 (19)	−0.0217 (3)	0.33260 (16)	0.0334 (6)	
H19	1.0800	0.0170	0.2853	0.040*	

### Atomic displacement parameters (Å^2^)

**Table:**

	*U*^11^	*U*^22^	*U*^33^	*U*^12^	*U*^13^	*U*^23^
Au	0.02340 (8)	0.02290 (8)	0.01865 (7)	0.000	0.00992 (5)	0.000
I1	0.0565 (2)	0.02093 (14)	0.0547 (2)	0.000	0.02773 (17)	0.000
P1	0.0234 (3)	0.0198 (3)	0.0171 (3)	0.0003 (2)	0.0095 (3)	0.0002 (2)
O1	0.0317 (12)	0.0451 (14)	0.0453 (14)	−0.0044 (10)	0.0176 (10)	−0.0003 (11)
C1	0.0315 (14)	0.0184 (13)	0.0201 (12)	0.0036 (10)	0.0081 (11)	0.0012 (10)
C2	0.0316 (16)	0.0266 (15)	0.0329 (15)	−0.0010 (12)	0.0070 (12)	−0.0041 (12)
C3	0.048 (2)	0.0341 (18)	0.043 (2)	−0.0060 (15)	0.0031 (17)	−0.0143 (14)
C4	0.070 (3)	0.0409 (19)	0.0264 (17)	0.0090 (17)	0.0058 (17)	−0.0148 (14)
C5	0.065 (2)	0.0395 (19)	0.0250 (15)	0.0110 (17)	0.0197 (15)	−0.0016 (13)
C6	0.0443 (18)	0.0297 (16)	0.0256 (14)	−0.0004 (12)	0.0167 (13)	−0.0037 (11)
C7	0.0307 (17)	0.0416 (19)	0.055 (2)	−0.0137 (15)	0.0108 (15)	−0.0047 (17)
C8	0.0232 (13)	0.0254 (13)	0.0177 (11)	−0.0004 (11)	0.0097 (10)	−0.0018 (10)
C9	0.0330 (16)	0.0359 (17)	0.0383 (16)	0.0090 (13)	0.0189 (14)	0.0083 (13)
C10	0.0277 (16)	0.064 (2)	0.0415 (19)	0.0130 (16)	0.0147 (15)	0.0103 (17)
C11	0.0278 (16)	0.065 (2)	0.0333 (17)	−0.0088 (15)	0.0149 (14)	−0.0056 (15)
C12	0.0380 (18)	0.0402 (17)	0.0342 (16)	−0.0158 (14)	0.0177 (14)	−0.0038 (13)
C13	0.0285 (14)	0.0298 (15)	0.0263 (13)	−0.0023 (12)	0.0106 (11)	0.0024 (11)
C14	0.0188 (12)	0.0208 (12)	0.0225 (12)	−0.0012 (10)	0.0075 (10)	0.0006 (10)
C15	0.0258 (14)	0.0359 (16)	0.0305 (14)	0.0028 (12)	0.0124 (12)	0.0083 (12)
C16	0.0251 (15)	0.0381 (17)	0.0478 (19)	0.0041 (13)	0.0102 (14)	0.0174 (15)
C17	0.0318 (17)	0.0269 (17)	0.059 (2)	0.0096 (13)	0.0052 (16)	0.0034 (15)
C18	0.052 (2)	0.042 (2)	0.053 (2)	0.0166 (17)	0.0151 (18)	−0.0146 (17)
C19	0.0378 (17)	0.0335 (16)	0.0276 (14)	0.0088 (13)	0.0130 (13)	−0.0011 (12)

### Geometric parameters (Å, º)

**Table:**

Au—P1^i^	2.3200 (6)	C9—C10	1.381 (4)
Au—I1	2.7188 (3)	C9—H9	0.9400
Au—P1	2.3200 (6)	C10—C11	1.370 (5)
P1—C14	1.816 (3)	C10—H10	0.9400
P1—C1	1.837 (3)	C11—C12	1.369 (5)
P1—C8	1.831 (3)	C11—H11	0.9400
O1—C7	1.200 (4)	C12—C13	1.388 (4)
C1—C6	1.384 (4)	C12—H12	0.9400
C1—C2	1.407 (4)	C13—H13	0.9400
C2—C3	1.398 (4)	C14—C15	1.388 (4)
C2—C7	1.467 (5)	C14—C19	1.387 (4)
C3—C4	1.383 (6)	C15—C16	1.385 (4)
C3—H3	0.9400	C15—H15	0.9400
C4—C5	1.369 (5)	C16—C17	1.368 (5)
C4—H4	0.9400	C16—H16	0.9400
C5—C6	1.392 (4)	C17—C18	1.378 (5)
C5—H5	0.9400	C17—H17	0.9400
C6—H6	0.9400	C18—C19	1.384 (4)
C7—H7	0.9400	C18—H18	0.9400
C8—C9	1.396 (4)	C19—H19	0.9400
C8—C13	1.392 (4)		
			
P1—Au—I1	115.755 (16)	C10—C9—C8	120.5 (3)
P1^i^—Au—P1	128.49 (3)	C10—C9—H9	119.8
P1^i^—Au—I1	115.755 (16)	C8—C9—H9	119.8
C14—P1—C1	104.04 (12)	C9—C10—C11	121.2 (3)
C14—P1—C8	102.94 (12)	C9—C10—H10	119.4
C1—P1—C8	104.32 (12)	C11—C10—H10	119.4
C14—P1—Au	115.85 (9)	C12—C11—C10	119.1 (3)
C1—P1—Au	121.94 (8)	C12—C11—H11	120.4
C8—P1—Au	105.63 (8)	C10—C11—H11	120.4
C6—C1—C2	118.6 (3)	C11—C12—C13	120.7 (3)
C6—C1—P1	120.6 (2)	C11—C12—H12	119.6
C2—C1—P1	120.8 (2)	C13—C12—H12	119.6
C3—C2—C1	119.5 (3)	C12—C13—C8	120.7 (3)
C3—C2—C7	117.3 (3)	C12—C13—H13	119.7
C1—C2—C7	123.1 (3)	C8—C13—H13	119.7
C4—C3—C2	120.6 (3)	C15—C14—C19	118.6 (3)
C4—C3—H3	119.7	C15—C14—P1	121.7 (2)
C2—C3—H3	119.7	C19—C14—P1	119.4 (2)
C5—C4—C3	119.8 (3)	C14—C15—C16	120.6 (3)
C5—C4—H4	120.1	C14—C15—H15	119.7
C3—C4—H4	120.1	C16—C15—H15	119.7
C4—C5—C6	120.3 (3)	C17—C16—C15	120.0 (3)
C4—C5—H5	119.8	C17—C16—H16	120.0
C6—C5—H5	119.8	C15—C16—H16	120.0
C5—C6—C1	121.1 (3)	C16—C17—C18	120.2 (3)
C5—C6—H6	119.5	C16—C17—H17	119.9
C1—C6—H6	119.5	C18—C17—H17	119.9
O1—C7—C2	125.5 (3)	C19—C18—C17	120.0 (3)
O1—C7—H7	117.2	C19—C18—H18	120.0
C2—C7—H7	117.2	C17—C18—H18	120.0
C9—C8—C13	117.7 (2)	C18—C19—C14	120.5 (3)
C9—C8—P1	117.6 (2)	C18—C19—H19	119.8
C13—C8—P1	124.4 (2)	C14—C19—H19	119.8

Symmetry code: (i) −*x*+2, *y*, −*z*+1/2.
